# IgE to wheat, prick test, and Patch test among children with celiac disease

**DOI:** 10.1186/s12887-024-04844-6

**Published:** 2024-05-28

**Authors:** Marjaneh Khalighi, Hazhir Javaherizadeh, Mehran Hakimzadeh, Mitra Ahmadi, Mehdi Torabizadeh, Abbas Fayezi

**Affiliations:** 1 Alimentary Tract Research Center, Clinical Sciences Research Institute, Imam Khomeini Hospital, Ahvaz Jundishapur University of Medical Sciences, Ahvaz, Iran; 2 Department of Allergy and Clinical Immunology, School of Medicine, Ahvaz Jundishapur University of Medical Sciences, Ahvaz, Iran

**Keywords:** Celiac disease, Diarrhea, Patch test, Prick test

## Abstract

**Introduction and aim:**

Celiac disease is one of the most common autoimmune disorders. This study aimed to evaluate the relationship between celiac disease and wheat sensitization.

**Subjects and methods:**

In the current study, children aged < 18 years with confirmed celiac disease were included. Data were analyzed using SPSS.

**Results:**

Gastrointestinal problems were the most common indication for evaluation in terms of celiac disease. Prick and patch tests were positive in 43.4% and 34% respectively.

**Conclusion:**

Prick test and patch test for wheat sensitization were positive in about 30–45% of the children for celiac disease.

## Introduction and aim

Celiac disease is an immune enteropathy caused by gluten ingestion. Prevalence of celiac disease was increased [1]. Intestinal and extraintestinal presentation of celiac disease were reported. There are few case reports about the co-existence of celiac disease and wheat allergy [2, 3]—the current study aimed to evaluate a possible association between celiac disease and wheat sensitization.

## Subjects and methods

This cross-sectional study was carried out among children aged < 18 years old in children with a diagnosis of celiac disease in Abuzar Childen’s Hospital and Celiac registry of Imam Khomeini Hospital, Ahvaz-Iran. Children with different stages of celiac disease were included in our study. All children with confirmed celiac disease were considered. Diagnosis of celiac disease was made with Anti TTG evaluation and multiple biopsies from duodenum by pediatric gastroenterologists. Modified Marsh classification was used in our clinical setting to diagnose celiac disease [4]. Exclusion criteria were poor compliance with the prick-and-patch test and the use of drugs that interfere with the prick-and-patch test. Prick and patch tests were used for all children in our study [5, 6]. Skin prick test evaluates immediate IgE-mediated allergy. The healthcare person will gently prick a drop of allergen in the forearm or back and assess for redness and swelling after 15–20 min. Patch test was used to evaluate delayed-type hypersensitivity. Patches of allergen were taped to the patient’s back. After a few days, cases were assessed for reading of patch test. All participants were informed about the details of testing with the manual of testing in the local language.

Diagnosis using pathology was included in the current study. Marsh classification was used to diagnose celiac disease. IgE reaction with gluten was evaluated using the CAP method [7]. Age, sex, symptoms, prick, and patch test were analyzed. Correlations were done using Chi-square. SPSS ver was used for analysis. A T-test was used for the Mean with normal distribution. Mann-Whitney was used for the Mean with abnormal distribution. *P* value < 0.05 was considered significant.

## Results

In the current study, 53 (m = 20, f = 33) cases were included ( Table 1). The most common reason for the investigation was gastrointestinal complaints (75.5%) followed by growth retardation (17%). The most common complaint among gastrointestinal problems was stomachache (Table 1). The most common grade was 3b in 71.7% of children. Prick tests and patch tests were positive in 43.4% and 34% of the patients with celiac disease( Table 1). Anemia was the most commonly associated symptom (Table 1). Of the cases, one case had respiratory problems. Diarrhea was seen in 12(22.6%); nausea in 3(5.7%); stomachache in 17(32.1%); constipation in 8(15.1%) of cases. Of the cases, 13(24.5%) had no gastrointestinal symptoms.

**Table 1:** Demographic and clinical manifestation and complication among children

Variables		Frequency	Percent
**Sex, N (%)**	Female	33	62.3
	Male	20	37.7
**Feeding 6-month, N (%)**	Formula	7	13.2
	Breastfeeding	39	73.6
	Breastfeeding + Formula	3	5.7
	Breastfeeding + Cow Milk	1	1.9
**The reason for the investigation, N (%)**	Diabetes	3	5.7
	GI	40	75.5
	Growth retardation	9	17.0
	Poor weight gain	1	1.9
**Accompanying symptoms, N (%)**	Anemia	10	18.9
	Anemia and hypothyroidism	1	1.9
	Anemia and Dental problems	1	1.9
	Aphthous	2	3.8
	Diabetes	1	1.9
	Growth retardation	7	13.2
	Herpetiform dermatitis	1	1.9
	Hypothyroid	2	3.8
	None	24	45.3
	Poor weight gain	1	1.9
	Psoriasis	1	1.9
	Weight loss	1	1.9
	Aphthous stomatitis	1	1.9
**Pathology, N (%)**	1	1	1.9
	2	9	17.0
	+ 3	1	1.9
	3a	2	3.8
	3b	38	71.7
	3c	2	3.8
**Patch test, N (%)**	+ 1	8	15.1
	+ 2	6	11.3
	+ 3	1	1.9
	Neg	35	66.0
**Prick test (wheal), N (%)**	+ 1	1	1.9
	+ 3.5	1	1.9
	+ 3	15	28.3
	+ 4	2	3.8
	+ 3.5	1	1.9

Laboratory findings among children with celiac disease are shown in Table 2.

As seen in Table 3, there is no significant correlation between patch test response and age at diagnosis( *p* = 0.35), total IgE level (*p* = 0.517), eosinophil count (*p* = 0.39), and anti-TTG level(*p* = 0.846). No significant correlation was seen between prick test response, eosinophil count, tissue transglutaminase level, and IgE to wheat( *p* > 0.05).

**Table 2:** Laboratory findings among children with celiac disease

Variables	Minimum	Maximum	Mean ± SD
**Age at diagnostic (Year), Mean ± SD**	2	18	6.94 ± 3.36
**Time to diagnostic (Year), Mean ± SD**	0.25	10	1.83 ± 1.63
**Total IgE (mg/dl), Mean ± SD**	2.0	2600	312.58 ± 611.18
**EOS (N/dl), Mean ± SD**	35	1094	529.32 ± 251.45
**Anti-TTG (RU/mL), Mean ± SD**	20.0	1238	230.09 ± 22.94
**IgE wheat (mg/dl), Mean ± SD**	0	0.45	0.05 ± 0.075
**WBC (µL), Mean ± SD**	2700	14,800	7212.08 ± 1958.58
**Hb (gr/dl), Mean ± SD**	6.4	5400	113.93 ± 740.06

Immunoglobulin E (IgE), Eosinophils (EOS), Tissue Transglutaminase (TTG), White Blood Count (WBC), Hemoglobin (Hb)

**Table 3:** Comparison between Prick test and Patch test results in terms of laboratory findings

Variables	Patch Test			*P*-Value	Prick Test				*P*-Value
	Negative	+ 1	+ 2		Negative	+ 3	+ 3.5	+ 4	
**Age at diagnostic (Year), Mean ± SD**	7.09 ± 2.98	8.13 ± 4.91	5.33 ± 3.88	0.35	6.90 ± 3.75	7.47 ± 3.292	6.50 ± 2.12	4.5 ± 0.7	0.843
**Time to diagnostic (Year), Mean ± SD**	2.01 ± 1.9	1.75 ± 0.7	0.81 ± 0.37	0.128	2.09 ± 2.07	1.6429 ± 0.74495	1 ± 0.02	1.5 ± 0.71	0.734
**Total IgE (mg/dl), Mean ± SD**	407.77 ± 728.00	166.97 ± 234.03	130.83 ± 86.40	0.517	334.19 ± 661.87	338.887 ± 671.1873	380 ± 56.56	243.5 ± 299.11	0.568
**EOS (N/dl), Mean ± SD**	505.23 ± 260.16	657.12 ± 238.04	463.67 ± 256.38	0.39	542.97 ± 260.44	491.87 ± 251.377	518 ± 53.74	494.5 ± 573.46	0.957
**Anti TTG (RU/mL), Mean ± SD**	240.72 ± 244.96	203.00 ± 100.96	164.83 ± 56.64	0.846	240.18 ± 241.60	208.867 ± 168.3805	138 ± 123.03	250 ± 70.71	0.435
**IgE wheat (mg/dl), Mean ± SD**	0.06 ± 0.081	0.047 ± 0.042	0.051 ± 0.08	0.5	0.04 ± 0.05	0.0660 ± 0.05356	0.24 ± 0.29	0.015 ± 0.007	0.593
**WBC (µL), Mean ± SD**	6925.43 ± 1736.18	7980.00 ± 1353.64	6541.67 ± 906.87	**0.044**	6933 ± 1474.81	7262.00 ± 2096.753	5930 ± 523.25	7750 ± 1909.19	0.469
**Hb (gr/dl), Mean ± SD**	12.32 ± 1.05	12.26 ± 2.74	12.58 ± 1.20	0.883	12.23 ± 1.02	12.907 ± 1.3371	12.8 ± 0.56	8.75 ± 3.32	0.08

## Discussion

In our study, prick, and patch tests for wheat sensitization were positive in 43.4% and 34%, respectively. In another study by Jafari et al., among 44 patients with celiac disease, 22 had at least one positive skin prick test for food allergen [8]. Wheat allergy was seen in 18.2% of the children with celiac disease in their study [8]. In another study from Brazil, wheat allergy was seen in 4% of children with celiac disease [9]. Other studies reported 8.3–11.9% of wheat allergies among children with Celiac disease [10–12].

In a systematic review, authors recommended screening of allergies in patients who have symptoms after introducing a gluten-free diet [13]. Wheat allergy is the most common allergy among patients with CD, especially in children [14]. Wong et al. reported a girl with CD and IgE-mediated wheat allergy [15]. The positive association between celiac disease and IgE sensitization to some food was also reported [16]. In the study by Spoerl et al., a positive association between celiac disease and wheat allergy was not found [17].

It seems that the frequency of wheat sensitization in children with celiac disease in our country was higher than in other studies.

Anemia was seen in 18.9% of the children. In another study, iron deficiency anemia was seen in 30% of children with celiac disease [18].

As mentioned above, the frequency of wheat sensitization was higher than in other reports, and this may be due to differences in ethnicity and type of evaluation. However, due to the high frequency of wheat allergy in our community, other studies are recommended.

### Limitation

Single-center study and lack of local data for wheat sensitization in our geographic location.

## Conclusion

The frequency of wheat sensitization among children with celiac disease was higher than in other studies. More studies are recommended.

## Data Availability

Data is available with a reasonable request from the corresponding author.
